# Encapsulation of Acid Whey in Alginate Microspheres for Application in Skin Microbiome-Friendly Topical Formulations: Optimization Through a Design of Experiments Approach

**DOI:** 10.3390/molecules30193907

**Published:** 2025-09-28

**Authors:** Elżbieta Sikora, Anna Łętocha, Alicja Michalczyk, Agnieszka Kozik

**Affiliations:** 1 Department of Organic Chemistry and Technology, Faculty of Chemical Engineering and Technology, Cracow University of Technology, Warszawska 24, 31-155 Cracow, Poland; agnieszka.kozik@student.pk.edu.pl; 2 Lukasiewicz—Research Network-Institute of Industrial Organic Chemistry, Annopol 6, 03-236 Warsaw, Poland; alicja.michalczyk@ipo.lukasiewicz.gov.pl

**Keywords:** whey, microspheres, skin microbiome, optimization

## Abstract

Skin microbiome-friendly preparations are gaining increasing popularity in the cosmetics and pharmaceutical industries. Fermented plants, lysates, and heat-treated products are used as probiotic ingredients in cosmetics. This is due to the presence of *Lactobacillus* bacteria, such as acid or acid-rennet whey, which are natural probiotics that can positively impact the skin microbiome. However, due to technological difficulties, the direct use of whey as a cosmetic ingredient is limited. An optimized emulsification method was used to obtain alginate microspheres as carriers of whey. The process parameters were optimized using the Design of Experiments (DoEs) methodology. The effect of three key variables, including the type of probiotic raw material (whey from 1—cows, 2—goats, and 3—mixed), the alginate-to-raw material ratio (1–3%), and sonication time (0.5–1.5 min), on parameters such as encapsulation efficiency, bacterial survival, viscosity, and microspheres size was analyzed. The results obtained demonstrated that the optimal process parameters were the sonication time of 0.5 min and the alginate-to-whey mass ratio of 1.5% for all types of whey material studied. However, the most important factor influencing the properties and functionality of the microspheres was sonication time. The optimized whey-loaded microspheres were incorporated into a preservative-containing emulsion system, in which the viability of whey-derived bacteria was monitored over time. The whey encapsulation process effectively maintained the bacteria’s probiotic properties, protecting their viability despite the presence of preservatives (at a level of 4.92 ± 0.9 log CFU/g after 30 days of formulation storage), thus confirming the feasibility of incorporating liquid whey into skincare formulations.

## 1. Introduction

Currently, special attention is being paid to the crucial role that the skin microbiome (SM) plays in its ability to function. The demand for microbiome-compatible skincare formulations is increasing within the cosmetics industry, driven by growing consumer awareness of skin’s microbiome health [1,2,3,4,5]. Recent studies highlight the importance of the microbiome in the context of skin cancers and emphasize its interactions with the immune system in both health and disease [6,7,8,9]. An imbalance of the skin microbiome, known as dysbiosis, can contribute to the development of various skin disorders [10]. Substances from the pre- and probiotics group, which have been successfully used for years as ingredients of dietary supplements and foods, are now also recognized as beneficial for skin dysbiosis [11,12].

In the case of cosmetics, the direct use of live probiotic bacteria is significantly limited due to the necessity of preservatives, which are responsible for maintaining the appropriate, long-term quality of cosmetics (in accordance with EU Directive No. 1223/2009) [13]. Therefore, current cosmetic formulations of probiotic ingredients from plant ferments, lysates, and tyndallized products are being used instead of probiotic bacteria [14,15]. An interesting source of probiotics (including live cultures of lactic acid bacteria) is milk whey, a byproduct of the dairy industry generated during the production of cheese and cottage cheese from different kinds of milk: cow’s, goat’s, or buffalo’s milk [16]. Depending on the technique used for cheese production, a distinction is made between acid whey, rennet whey, and acid-rennet whey. Producing 1 kg of cheese from cow’s milk produces approximately 9 kg of whey, representing over 160 million tons of production residue annually (65% of which is rennet whey and 35% of which is acid whey) [17,18].

In the past, whey was considered a waste byproduct, but in recent decades, scientific evidence has demonstrated that whey has biological, nutritional, and technological value [19]. It is a cloudy, yellowish liquid composed of lactose, lipids, minerals, and an excellent protein composition dissolved in water. Whey proteins are obtained by precipitating casein during pH regulation of milk and include primarily β-lactoglobulin (48–58%), α-lactalbumin (13–19%), and immunoglobulins (8–12%), as well as serum albumin (5–6%), lactoferrin (2%), lactoperoxidase (0.5%), glycomacropeptide (12–20%), lysozyme, lactoferricin, and cytokines [20,21,22]. Products currently obtained from whey include animal feed, microbial biomass, baker’s yeast, functional meals, whey protein concentrate (WPC), edible films and coatings, lactic acid, alcoholic beverages, sports drinks, and other biochemical or biofuels [16,23]. WPC and WPI (whey protein isolate) are two popular types of whey protein products. WPC contains 60–80% protein, while WPI contains 85–95% protein. In addition, whey protein hydrolysate (WPH) represents an even more purified form of whey protein. Whey protein products are widely used in the food industry as emulsifiers, gelling agents, and foaming agents [24,25]. Apart from their application as functional foods with potential health benefits, whey can also be beneficial from a cosmetic perspective [20,26,27]. For example, the bioactive peptides produced during whey fermentation can increase the body’s glutathione content and enhance free radical scavenging activity, resulting in antioxidant effects [28,29]. Additionally, whey proteins effectively inhibit skin irritation and help reduce redness, accelerating skin regeneration after exposure to various stressors, such as UV radiation. They also influence skin pigmentation and epidermal thickness, acting as inhibitors of skin aging [20]. One of the most valuable whey proteins is lactoferrin, which exhibits antimicrobial, anti-inflammatory, and antioxidant properties [30]. Some scientific studies have indicated that oral supplementation with whey protein enriched in lactoferrin significantly reduces skin imperfections [31]. Whey has also been identified as a potential fermentation medium for the production of postbiotics [32]. There are some cosmetic products on the market utilizing rennet whey as the aqueous phase and as a source of bioactive compounds such as peptides, lactose, and whey proteins [33].

While rennet whey is used in cosmetics as a substitute for water in the aqueous phase, acid whey, due to its content of *Lactobacillus* bacteria, may serve as a natural probiotic, positively influencing the skin microbiome. For example, cow whey contains 5.6 log CFU/g mesophilic and 4.7 log CFU/g thermophilic *Lactobacillus* bacteria, while buffalo whey contains significantly higher amounts, at 7.0 log CFU/g and 8.5 log CFU/g, respectively [34].

It is worth emphasizing that the management of food processing residues, such as whey, as a full-value natural cosmetic raw material, is well aligned with the concept of sustainable development and the growing demand for eco-friendly cosmetic products.

However, due to limitations, the direct incorporation of live probiotic bacteria or raw materials containing them into cosmetics remains problematic. Probiotics must possess specific properties, such as good stability, resistance to high temperatures, and adequate shelf life [14,35]. In cosmetic products, microbiological safety requirements necessitate the use of preservatives [36]. In recent years, various microencapsulation techniques have been applied to protect probiotic bacteria from adverse environmental conditions that compromise microbial viability [25,37,38,39]. Among these approaches, carriers in the form of polymer microspheres have been used to preserve the viability of probiotic bacteria in cosmetic formulations containing preservatives [40,41,42].

In this study, an optimized emulsification method was employed to produce alginate microspheres as carriers for acid whey, intended for potential use as an active probiotic raw material in cosmetic formulations. The effect of emulsification parameters on the physicochemical and microbiological properties of whey-loaded microspheres was evaluated. A statistical Design of Experiments (DoEs) approach was applied to optimize the process parameters, and the resulting optimal microspheres were incorporated into a cosmetic formulation, in which the viability of whey-derived bacteria was monitored over time.

To our knowledge, this is the first study to combine acid whey as a cosmetic raw material in the context of probiotic actions with alginate-based encapsulation, while simultaneously evaluating the impact of process parameters on both physicochemical and microbiological properties. Unlike previous research that typically employs isolated probiotic strains or commercial whey protein concentrates, our method leverages the native microbiota of goat and/or bovine-derived acid whey; this has not been extensively explored in encapsulation systems. This integrative approach could lead to significant advancements in the field of probiotic delivery systems in the case of cosmetic applications.

## 2. Results and Discussion

Studies in the literature indicate that whey proteins (WPC and WPI) have been used as carriers in co-encapsulation processes with alginates [43,44,45,46], as well as ingredients enhancing the functional properties of cosmetics. These proteins exhibit foaming capacity and emulsion-stabilizing abilities [47]. Moreover, they are associated with a range of health-promoting effects, including antimicrobial, anticancer, immunostimulatory, and antioxidant effects [48,49,50,51]. To date, however, there have been no reports of the use of unprocessed whey (i.e., unpasteurized and non-lyophilized) as a raw cosmetic material that simultaneously serves as a source of live probiotic bacteria and as a functional component in skin care products. These limitations arise from the technological challenges related to the direct incorporation of whey-derived probiotics into cosmetic formulations. To address these challenges and enable the use of probiotic-containing whey as an active ingredient in cosmetics, alginate microspheres were used as a carrier system.

**Figure 1:**
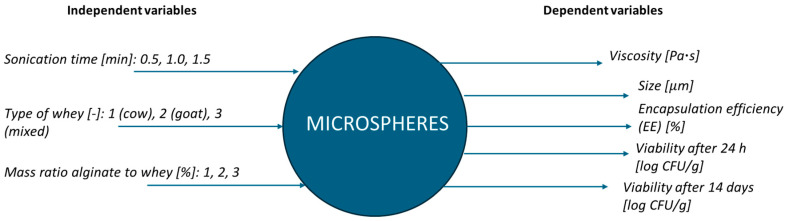
Visual summary of the optimization workflow.

Mathematical methods were used to optimize the composition of whey-loaded microspheres. The ranges of the independent process variables are shown in Figure 1.

Table 1 presents the detailed process of composition parameters, along with the results of the physicochemical and microbiological analyses of the whey-loaded alginate microspheres.

**Figure 2:**
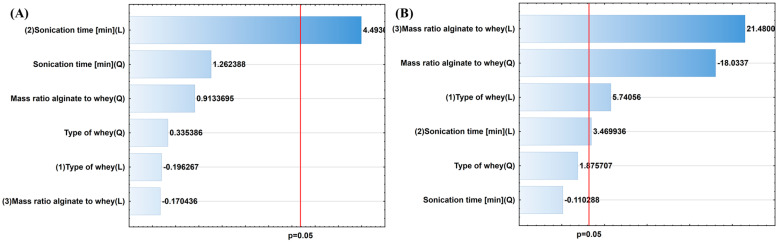
Pareto charts for physicochemical properties of prepared compositions: (**A**) microspheres size after 24 h [µm] and (**B**) viscosity [Pa∙s].

**Figure 3:**
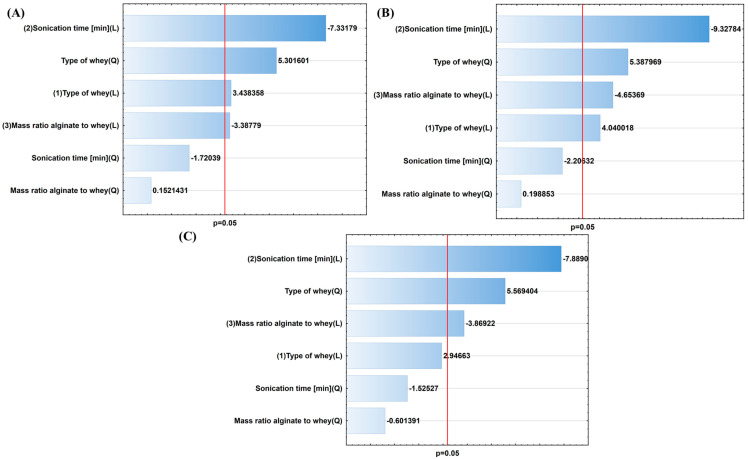
Pareto charts for microbiological properties of prepared compositions: (**A**) encapsulation efficiency [%], (**B**) viability after 24 h, and (**C**) 14 days [CFU/g].

The first stage of statistical analysis involved Pareto charts (Figure 2 and Figure 3), which were used to assess whether the input parameters had statistically significant effects on the output parameters. The red line indicates statistical significance at *p* < 0.05. Sonication time was a significant factor influencing microsphere size, with a positive effect on their diameter. As such, extending the sonication time reduced their size. This can be explained as follows: longer periods of ultrasonic exposure increase the number of cavitation cycles, and therefore, the frequency and intensity of mechanical interactions. As a result, the particles undergo progressive fragmentation into increasingly smaller fractions, and their average size systematically reduces with increasing sonication time [52].

The analysis of factors influencing viscosity revealed a statistically significant effect of the alginate-to-whey mass ratio (both linear and quadratic), as well as the type of whey and sonication time. The most significant parameter was the alginate-to-whey mass ratio, which can be explained by the fact that as the alginate content in the system increases, the density of the network of bonds between the polysaccharide and whey molecules increases. This leads to more intense electrostatic interactions and the formation of a more ordered colloidal structure, which consequently results in increased viscosity [53].

Microbiological parameters included EE and microbial viability over time. EE reflects the percentage of viable microorganisms effectively trapped in the carrier system relative to the amount initially added. In the analysis of these parameters, sonication time proved to be a key factor, demonstrating a negative impact on both EE and the viability of microorganisms. This indicates that extending sonication time adversely affects these parameters. This is consistent with studies by other research groups, which identified that extending ultrasound exposure reduces the number of microorganisms [54]. Inactivation of microorganisms by ultrasound occurs due to the thinning of cell membranes, the rupture and shearing of cell walls, formation of hot spots, production of free radicals, and DNA damage [55,56,57].

The complete general regression equation is presented below (Equation (1)), incorporating all independent parameters. The coefficients of Equation (1) are summarized in Table 2.

y = a + bx_1_ + cx_1_^2^ + dx_2_ + ex_2_^2^ + fx_3_ + gx_3_^2^ (1)

where x_1_—type of whey; x_2_—sonication time; x_3_—alginate-to-whey mass ratio.

**Figure 4:**
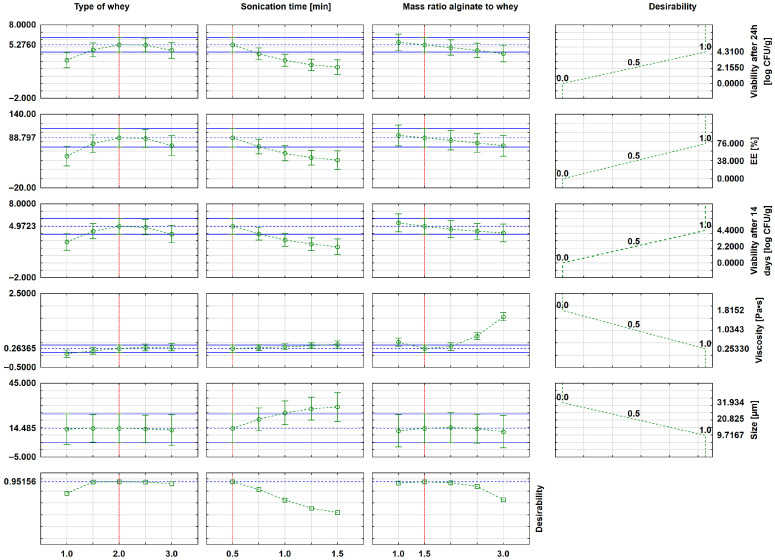
Approximation profiles in the DOE approach showing the influence of independent parameters on dependent variables.

During the next stage of the statistical analysis, approximation profiles were generated. As presented in Figure 4, these profiles indicate the optimal ranges of input parameters that lead to favorable results for the output variables. The most desirable values are the smallest particle size (to enable effective bacterial encapsulation, i.e., no less than 1 μm), low viscosity—which is crucial for microsphere dispersions as it facilitates sedimentation and enables efficient separation from the microdispersion during centrifugation—and the highest values of microbiological parameters (EE and bacterial viability over time).

The analysis of the approximation profiles (Figure 4) shows that the microspheres with the smallest size (≈10 μm) and viscosity (0.263 Pa∙s) and the highest EE (78%), viability after 24 h (4.31 log CFU/g) and 14 days (4.4 log CFU/g) were obtained at the lowest sonication time (0.5 min), 1.5% alginate-to-whey ratio and the second type of the whey (goat whey). The graphs also show that microbiological parameters decrease with increasing sonication time and higher alginate-to-whey ratios, which can be explained by both the mechanical effects of ultrasound and the increased density of the polysaccharide matrix, limiting microbial survival. The better results obtained for goat whey are due to the lower number of starter microorganisms subjected to encapsulation, which translates into a higher EE. It should be emphasized, however, that such a carrier can also be effectively applied to the encapsulation of other probiotic sources, opening up prospects for broader applications in cosmetic technology.

Based on the results of the DoE, optimal whey-loaded alginate microspheres were prepared (Table 3). These microspheres were characterized by low viscosity (<1 Pa·s), a small yet sufficient particle size (>1 µm), high EE (>70%), and high bacterial viability over time.

**Figure 5:**
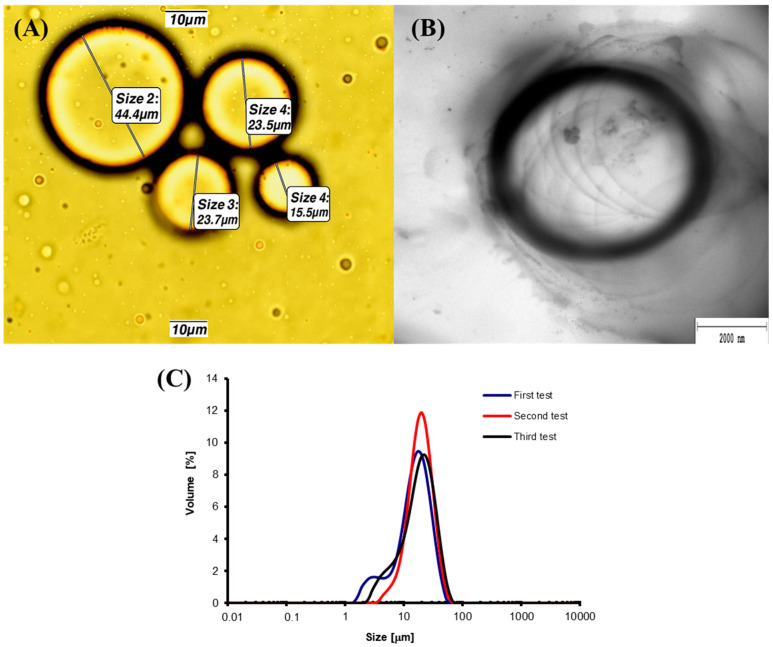
Size and morphology of optimal goat whey–alginate microspheres obtained under process conditions, including a sonication time of 0.5 min and an alginate-to-whey mass ratio of 1.5%: (**A**) optical micrograph, (**B**) transmission electron micrograph, and (**C**) laser diffraction measurements.

The size and morphology of the obtained microspheres were analyzed using optical microscopy and transmission electron microscopy (TEM) (Figure 5). Furthermore, the particle size distribution was determined using laser diffraction (Figure 5C).

The average diameter of the microspheres determined by laser diffraction was 22.9 ± 1.3 μm, showing good agreement with the results obtained via optical microscopy, where the mean value from 200 measurements was 23.36 ± 2.25 μm.

The microspheres obtained in our study exhibited smaller diameters compared to those reported by other research groups. For instance, in the study by Zanjani et al. [58], where an emulsification technique was used to encapsulate *Lactobacillus casei* ATCC 39392 in alginate microspheres, the resulting particles ranged in size from 90 ± 1.69 μm to 125 ± 1.81 μm.

In a study by Annan et al. [59], *Bifidobacterium adolescentis* 15703T was encapsulated in alginate microspheres, yielding a mean particle size of 53.1 ± 10.3 μm. The smaller microsphere sizes obtained in our study may represent a beneficial feature enhancing their applicability in cosmetic formulations. The TEM image of microdispersion confirmed the spherical morphology of the microspheres and their micrometer-scale dimensions. These findings are consistent with previous studies, including the encapsulation of *Lactobacillus casei* ATCC 393 in alginate microspheres [60].

**Figure 6:**
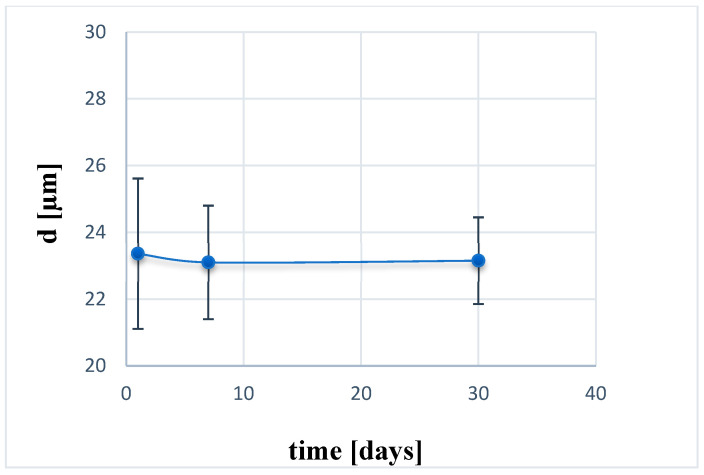
Droplet size of goat whey-loaded microspheres as a function of time at 25 °C (mean value ± standard deviation (SD), n = 3).

Additionally, as shown in Figure 6, the particle size remained virtually unchanged over 30 days of storage at 25 °C, confirming the dimensional stability of the microspheres.

The optimized microspheres loaded with goat whey were introduced into a cosmetic formulation, and the viability of whey bacteria was assessed immediately after the production of the cosmetic emulsion and after 7 and 30 days before being compared to the control formulation (containing unencapsulated whey) (Table 4).

The initial density of probiotic bacteria in whey was 7.9 log CFU/g. When assessing bacterial viability in unencapsulated whey, the bacterial count in the cosmetic formulation initially decreased by 1.52 log. After one week of storage, the number of microorganisms increased slightly, as the log reduction was lower than immediately after preparation, reaching 1 log CFU/g. However, after 30 days of storage, the complete reduction of probiotic microorganisms was observed in the formulation (Table 4). This result also confirms the effectiveness of the applied preservative system, as the complete elimination of microorganisms after 30 days in the formulation without encapsulation indicates proper performance.

By contrast, in the case of the cosmetic product containing encapsulated goat whey, the initial density of probiotics in the microspheres was 6.35 log CFU/g. Immediately after emulsion preparation, a 1.55 log decrease in probiotic cell count was observed. Tests conducted after 7 and 30 days of storage showed a decrease in bacterial cell viability of 1.65 and 2.98 log, respectively (Table 4).

The use of alginate microspheres as a whey probiotic carrier allowed the survival of lactic acid bacteria in the formulation despite the presence of preservatives, while simultaneously protecting the system from uncontrolled microbial growth. The cosmetic formulation contained a preservative system which, in the case of unencapsulated whey, resulted in the complete reduction in probiotic microorganisms after 30 days of storage. In contrast, the encapsulation of whey within alginate microspheres enabled the survival of microorganisms over the same storage period, confirming both the efficacy of the preservative system and the protective function of the carrier in maintaining probiotic viability. The obtained results are consistent with our previous studies on the introduction of a probiotics-loaded carrier into the cosmetic formulation, which indicated the survival of probiotic bacteria for over 120 days [61].

As previously discussed, there are studies available in the literature on the application of whey, including whey proteins in particular, in cosmetic formulations [62]. According to data from the FDA’s Voluntary Cosmetic Registration Program (VCRP) for 2017 [62], hydrolyzed milk proteins were used in 189 formulations, with the majority of applications found in leave-on products.

In a study by Speer and Amin, the potential of an O/W emulsion containing whey protein concentrate, chitosan, and various oils on structural responses to temperature was investigated, and it was found that whey protein concentrate influenced the ability of the formulation to adapt to temperature changes, which is particularly important when developing cosmetics for specific climatic or geographical conditions [63]. The study by Hewitt et al. [64] also showed that the use of bioactive milk proteins, lactoferrin and whey proteins on a synthetic polymer scaffold (polycaprolactone) increased the growth, spread, and infiltration of keratinocytes and fibroblasts [65]. Although whey protein products are available on the market, the range of formulations containing liquid whey remains extremely limited. Studies by other research groups also indicate the use of liquid whey in cleansing preparations such as shampoos [47] and hydrogels [20].

However, to date, there have been no microbiome-friendly skin care formulations containing liquid acid whey as a probiotic, which underscores the innovative nature of this research. Cosmetics containing encapsulated acid whey, as a rich source of valuable actives and probiotic bacteria, align well with the concept of sustainable, natural, eco- and skin-friendly cosmetic products with proven safety.

## 3. Materials and Methods

### 3.1. Materials

Alginic acid sodium salt from brown algae, de Man-Rogosa-Sharpe (MRS) broth, MRS agar, and sodium citrate were purchased from Sigma Aldrich (Poznań, Poland). Calcium chloride was purchased from Avantor Performance Materials Poland S.A. (Gliwice, Poland). Cow and goat whey were purchased from Łomnicka Hala Mleczna (Łomnica, Poland) and Ekologiczne Gospodarstwo pod Kasztanem (Ludwinów, Poland), respectively. Caprylic/capric triglycerides, ECO-tween 80, and emulsifiers (glyceryl stearate, polyglyceryl-6 palmitate/succinate, cetearyl alcohol, sorbitan stearate, and sucrose cocoate) were kindly provided by Croda (Kraków, Poland). Meadowfoam seed oil was obtained from PipingRock Health Products (New York, NY, USA). Sodium levulinate, sodium anisate, and sodium benzoate were obtained from Evonik Industries AG (Essen, Germany). Xanthan gum was bought in Warchem (Zakręt, Poland). Deionized water was used as the solvent.

### 3.2. Encapsulation of Whey

**Figure 7:**
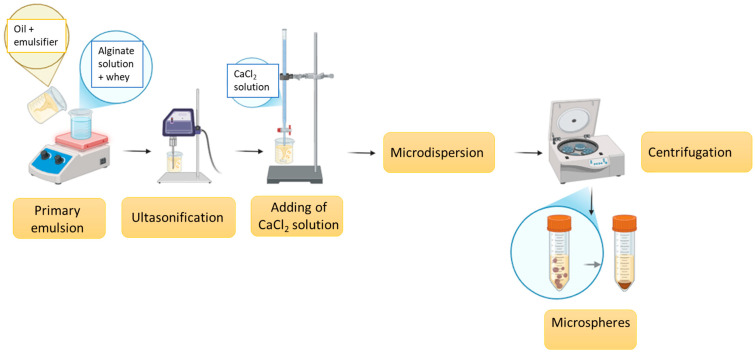
Schematic description of the encapsulation process of whey in the alginate microspheres.

Whey-loaded microspheres were prepared according to the modified methodology described by Łętocha et al. [60]. A schematic description of the encapsulation process is provided in Figure 7.

### 3.3. Optimization Process

The production of alginate microspheres was optimized using a mathematical Design of Experiments approach (Statistica version 13, StatSoft, Kraków, Poland). To obtain whey-loaded microspheres with desirable physicochemical and microbiological properties, a fractional factorial design 3^(K–p)^ was employed, where K denotes the number of variables and *p* is set to 1. The input factors included sonication time, type of whey, and the alginate-to-whey mass ratio. Experiments were performed in a randomized order.

#### 3.3.1. Physicochemical Properties

The influence of independent parameters on physicochemical properties—including the size and morphology of microspheres and viscosity of microdispersions—was determined.

##### Size and Morphology of the Microspheres

The droplet size of alginate microspheres was determined using a Motic B1 Advanced Series optical microscope (Hong Kong, China) equipped with a digital camera. The droplet diameter for each sample was calculated as the mean of 200 measurements ± SD. Additionally, the morphology of whey microdispersed samples was examined using a JEOL JEM 2100 HT transmission electron microscope (Jeol Ltd., Tokyo, Japan). Images were taken with a 4k (TVIPS) camera using EMMENU software version 1.0.4.0.9.87 (TVIPS GmbH, Gauting, Germany). For optimal microspheres, the mean droplet size and size distribution of the microsphere dispersions were also measured by laser diffraction using a Malvern Instruments MasterSizer 2000 (Worcestershire, UK) particle size analyzer at 25 °C. Samples were diluted with capric/caprylic triglycerides for measurement. Three replicates were performed for each sample.

##### Microdispersions Stability

The stability of microsphere dispersions at 25 °C was assessed by measuring droplet size (using an optical microscope) as a function of time after 24 h, 7, and 30 days.

##### Viscosity Measurement

The rheological properties of the microdispersions containing alginate spheres were evaluated using a rotational rheometer (Brookfield Model R/S Plus, Harlow, UK) at room temperature (25 °C), with shear rates up to 500 s^−1^ over a period of 60 s. Viscosity was calculated as the mean of three measurements ± S

#### 3.3.2. Microbiological Properties

The influence of independent parameters (sonication time, type of whey, and alginate-to-whey mass ratio) on encapsulation efficiency and bacterial viability in microspheres was determined.

##### Encapsulation Efficiency and Viability over Time

Encapsulation efficiency was assessed according to the methodology described by Guimarães et al. [66] and Łętocha et al. [60]. Briefly, 1 g of microspheres was dissolved in 9 mL of 0.2 mol·L^−1^ sterile sodium citrate solution (pH 6.0), and the solution was centrifuged. Serial dilutions were prepared, and the resulting solutions were plated on MRS agar. Viable cells were counted as the number of colonies obtained after 72 h incubation at 37 °C under aerobic conditions. All tests were performed in triplicate. The microencapsulation efficiency (EE [%]) of probiotic bacteria was calculated using Equation (2) and expressed as the percentage of log CFU·g^−1^.

EE (%) = (N/N_0_) × 100 (2)

where N represents the number of bacterial cells trapped in microspheres, and N_0_ indicates the number of free bacterial cells added during encapsulation [67,68].

### 3.4. Preparation of Formulation with Whey-Loaded Microspheres

The formulation was prepared according to the methodology described by Łętocha et al. [61]. Briefly, the oil and aqueous phases (containing emulsifiers) were separately heated to 70 °C, combined, and mixed using a mechanical stirrer (IKA C-MAG HS 7, Warsaw, Poland) at 600 rpm for 15 min. After the mixture was cooled below 40 °C, whey-loaded microspheres were incorporated, and mixing continued until the formulation reached 25 °C.

Table 5 presents the composition of the formulation containing whey-loaded microspheres. The composition is protected by patent application P.445990 [69].

### 3.5. Viability of Probiotics in Microencapsulated Whey in Skin Care Product

The viability of whey probiotic strains encapsulated in microspheres and incorporated into emulsions was evaluated according to the methodology described by Lasta et al. [70] and in our previous publication [61]. Optimal microspheres were used as the active ingredients in the emulsion. Bacterial viability was assessed immediately after incorporation into the cosmetic formulation and then after 7 and 30 days. Cell reduction was defined as the difference between the initial amount of free probiotic cells introduced into the cosmetic formulation, or the number of cells encapsulated in alginate microcapsules, and the number of viable cells remaining after storage. The experiments were conducted in duplicate, and the results were used to calculate the mean logarithmic reduction.

### 3.6. Statistical Analysis

All data concerning the mean droplet size, viscosity, and probiotic viability were presented as the means of three experiments ± SD. Differences between the calculated means of each individual group were determined by one-way ANOVA tests, using statistical software Statistica version 13 (StatSoft Company, Kraków, Poland). The value of *p* < 0.05 was considered statistically significant.

## 4. Conclusions

To sum up, the results of our study introduce a novel approach to skin microbiome-friendly products by utilizing unprocessed acid or acid-rennet whey—a dairy by-product—as a natural and sustainable source of probiotic microorganisms in cosmetic formulations. Unlike previous research, which typically employs isolated probiotic strains or commercial whey protein concentrates, our method leverages the native microbiota of goat- and/or bovine-derived whey. By optimizing the encapsulation process using alginate microspheres and applying the DoE methodology, a system was developed that ensures high encapsulation efficiency and microbial viability, even in the presence of preservatives. The incorporation of whey-loaded microspheres into a preservative-containing emulsion confirmed the system’s ability to maintain the viability of probiotic bacteria over time, thereby preserving their biological activity.

The results obtained demonstrate the potential of the optimized alginate microcapsule system to protect and maintain the biological functionality of entrapped whey (the source of lactic acid bacteria). This approach offers a novel and sustainable alternative to conventional probiotic ingredients, enabling the direct use of liquid and unprocessed whey in skin microbiome-friendly products. These findings contribute to the advancement of probiotic delivery systems in cosmetics and open new possibilities for valorizing dairy by-products in high-value products. However, further in vitro and in vivo studies are warranted to fully validate the functional benefits and long-term stability of this innovative approach.

## Figures and Tables

**Table 1:** Matrix of the DoE experiment for whey-loaded microspheres along with the experimental data obtained for the dependent variables.

Independent Variables				Dependent Variables				
Sample No.	Sonication Time [min]	Type of Whey *	Alginate-to-Whey Mass Ratio [%]	Viscosity [Pa∙s]	Size [µm]	EE ** [%]	Viability After 24 h [log CUF/g]	Viability After 14 Days [log CUF/g]
1	1	2	2	0.437 ± 0.18	24.4 ± 2.7	51 ± 2	2.89 ± 0.10	2.87 ± 0.12
2	0.5	1	1	0.345 ± 0.20	9.7 ± 2.8	48 ± 2	3.24 ± 0.10	3.12 ± 0.10
3	1.5	2	1	0.722 ± 0.12	26.7 ± 3.2	47 ± 2	2.66 ± 0.18	2.53 ± 0.12
4	1	3	1	0.626 ± 0.18	25.3 ± 4.5	48 ± 1	2.96 ± 0.16	2.86 ± 0.11
5	1.5	3	3	1.815 ± 0.15	23.0 ± 2.9	0	0	0
6	1.5	1	2	0.253 ± 0.09	31.9 ± 6.2	0	0	0
7	1	1	3	1.453 ± 0.10	21.7 ± 3.8	0	0	0
8	1	2	2	0.436 ± 0.12	24.0 ± 3.9	43 ± 1	2.43 ± 0.12	2.43 ± 0.15
9	0.5	3	2	0.435 ± 0.12	13.2 ± 2.2	68 ± 2	4.23 ± 0.14	3.39 ± 0.15
10	0.5	2	3	1.512 ± 0.08	15.4 ± 3.1	76 ± 1	4.31 ± 0.12	4.4 ± 0.14

* 1—cow whey; 2—goat whey; 3—mixed whey; ** encapsulation efficiency.

**Table 2:** Statistical ANOVA results.

Coefficients	Size [µm]			Viscosity [Pa∙s]			EE [%]			Viability After 24 h [log CFU/g]			Viability After 14 Days [log CFU/g]		
	Value	F-Test	*p*	Value	F-Test	*p*	Value	F-Test	*p*	Value	F-Test	*p*	Value	F-Test	*p*
a (stała)	−13.9580	-		1.47982	-		18.313	-		2.47750	-		1.92250	-	-
b	3.1767	0.03852	0.856944	0.42865	32.9540	0.010498	124.583	11.82231	0.041285	6.34833	16.32174	0.027287	6.91167	8.68263	0.060188
c	−0.8730	0.11248	0.759418	−0.07279	3.5183	0.157362	−28.312	28.10697	0.013099	−1.42250	29.03021	0.012526	−1.59750	31.01827	0.011424
d	40.7255	20.18788	0.020575	0.13198	12.0405	0.040348	−121.833	53.75519	0.005242	−7.70000	87.00858	0.002609	−6.29333	62.23728	0.004245
e	−13.1441	1.59362	0.296019	0.01712	0.0122	0.919145	36.750	2.95973	0.183848	2.33000	4.86785	0.114494	1.75000	2.32644	0.224606
f	9.2362	0.02905	0.875513	−2.28478	461.3921	0.000221	−7.917	11.47715	0.042844	−0.54833	21.65684	0.018716	−1.37500	14.97084	0.030543
g	−2.3775	0.83424	0.428396	0.69982	325.2133	0.000372	−0.813	0.02315	0.888730	−0.05250	0.03954	0.855092	0.17250	0.36167	0.589986
R^2^	0.887			0.996			0.973			0.981			0.975		
Adjusted R^2^	0.661			0.989			0.918			0.944			0.925		

**Table 3:** Input and output parameters for optimal whey-loaded alginate microsphere composition.

Sonication Time [min]	Type of Whey *	Alginate-to-Whey Mass Ratio [%]	Viscosity [Pa∙s]	Size [µm]	EE [%]	Viability After 24 h [log CFU/g]	Viability After 14 Days [log CFU/g]
0.5	2.0	1.5	0.437 ± 0.1	23.36 ± 2.25	72 ± 1	4.40 ± 0.32	4.31 ± 0.40

* 1—cow whey; 2—goat whey; 3—mixed whey.

**Table 4:** Viability of bacteria derived from goat whey in cosmetic formulations.

Sample No.	Immediately After Preparation		After 7 Days		After 30 Days	
	Cell Viability * [log CFU/g]	Log Reduction	Cell Viability [log CFU/g]	Log Reduction	Cell Viability [log CFU/g]	Log Reduction
Formulation with non-encapsulated whey	6.38 ± 1.1	1.52	6.90 ± 0.6	1.00	0	7.9
Formulation with whey-loaded microspheres	6.35 ± 0.7	1.55	6.25 ± 0.7	1.65	4.92 ± 0.9	2.98

* Cell viability is the mean value of two replicates ± SD.

**Table 5:** Compositions of the topical formulations [69].

Emulsion Phase	Component	Concentration [%]
Water phase	Water	77
	Xanthan gum	
	Sodium levulinate; sodium anisate	
	Sodium benzoate	
Oil phase	Meadowfoam seed oil	15
Emulsifiers	Sorbitan stearate; sucrose cocoate	7
	Glyceryl stearate; polyglyceryl-6 palmitate/succinate; cetearyl alcohol	
Active	Whey-loaded microspheres	

## Data Availability

The original contributions presented in this study are included in the article. Further inquiries can be directed to the corresponding authors.
